# Comprehensive Analysis of the Aberrance and Functional Significance of Ferroptosis in Gastric Cancer

**DOI:** 10.3389/fphar.2022.919490

**Published:** 2022-07-12

**Authors:** Jun Xiao, Lingyan Zheng, Jingfeng Liu

**Affiliations:** ^1^ Department of Gastrointestinal Surgery, Fujian Medical University Cancer Hospital, Fujian Cancer Hospital, Fuzhou, China; ^2^ Fujian Key Laboratory of Advanced Technology for Cancer Screening and Early Diagnosis, Fujian Medical University Cancer Hospital, Fujian Cancer Hospital, Fuzhou, China; ^3^ Department of Anus Intestine Surgery, Fuzhou Second Hospital Affiliated to Xiamen University, Fuzhou, China; ^4^ Department of Hepatopancreatobiliary Surgical Oncology, Fujian Medical University Cancer Hospital, Fujian Cancer Hospital, Fuzhou, China

**Keywords:** gastric cancer, ferroptosis, prognosis, tumor microenvironment, immune checkpoint blockade, drug sensitivity

## Abstract

**Objective:** Ferroptosis is a type of iron-dependent necrosis related to cancer. Nevertheless, the features of ferroptosis in gastric cancer (GC) remain poorly understood. This study conducted a systematic analysis of ferroptosis regulators in GC.

**Methods:** We gathered five GC cohorts, namely, TCGA-STAD, GSE84437, GSE62254, GSE26901, and GSE15459. Unsupervised clustering analysis was adopted to cluster GC patients into different ferroptosis subtypes based on ferroptosis regulators. Immune cell infiltration and hallmark pathway activity were estimated *via* ssGSEA. The ferroptosis index was developed with the PCA computational method. Response to chemotherapy agents and small molecular compounds was inferred *via* GDSC, CTRP, and PRISM projects. Two anti-PD-1 therapy cohorts were gathered and the potential of FPI in predicting immune response was assessed.

**Results:** Expression profiles, genetic mutations, DNA methylation, prognostic implications, and drug sensitivity of ferroptosis regulators were characterized in GC. Three ferroptosis subtypes were clustered with distinct prognosis, hallmark pathway activity, and tumor-infiltrating immune cells. Ferroptosis levels were quantified based on the expression of prognostic ferroptosis-related signatures. The significant relationships between FPI and clinicopathological characteristics were observed. Furthermore, high FPI was in relation to poor prognosis, inflamed tumor microenvironment (TME) as well as high sensitivity to chemotherapy agents (docetaxel and cisplatin), and CTRP- and PRISM-derived compounds. Also, FPI acted as a promising predictor of immune response.

**Conclusion:** Collectively, our findings identified a novel ferroptosis-based subtype classification of GC, and revealed the potential of ferroptosis in forming TME diversity and complexity, and guiding individualized treatment.

## Introduction

Gastric cancer (GC) represents a major malignant tumor globally, which ranks the fifth in cancer incidence and the third major cause of cancer-relevant deaths (Bray et al., 2018). As estimated, the 5-year survival rate is <20% (Cavatorta et al., 2018). GC exhibits distinct molecular heterogeneity with aggressive behaviors as well as therapy resistance (Yan et al., 2018). The conventional system for predicting survival outcomes, like histological grade and tumor staging, is of difficulty to cover the clinical diversity of GC (Shao et al., 2016). Hence, it is of urgency to discover more effective biomarkers for early diagnosis, therapy, and prognostic evaluation (Wang et al., 2021).

Ferroptosis has been described as an iron-dependent necrosis modulated by lipid peroxidation (Niu et al., 2022). Under the normal condition, polyunsaturated fatty acid is often oxidized by lipoxygenase like 12/15-lipoxygenase but instantly decreased by enzyme GPX4 as well as its cofactor GSH (Lee et al., 2020). Nevertheless, when GPX4 is suppressed or GSH is exhausted, lipid peroxide accumulates in cells, inducing lipid peroxidation-mediated cell deaths, named ferroptosis. Unlike apoptosis or necroptosis, ferroptosis does not depend on caspase or RIPK1 kinase activation. Recently, several studies have proposed the key roles of ferroptosis in modulating tumor development and drug resistance in GC. Stimulation of ferroptosis has been a promising strategy for treating GC (Liu et al., 2021). For instance, cancer-associated fibroblasts secreted micro-522, inhibits ferroptosis, and induces acquired chemotherapy resistance in GC (Zhang et al., 2020). Tanshinone IIA enhances ferroptosis in GC cells *via* p53-regulated SLC7A11 inactivation (Guan et al., 2020). Micro-375 weakens the stemness of GC cells *via* inducing ferroptosis (Ni et al., 2021). Nevertheless, the detailed features of ferroptosis in GC remain poorly understood. Herein, our study systematically analyzed ferroptosis regulators and characterized the potential of ferroptosis-based treatment in GC.

## Materials and Methods

### Gastric Cancer Datasets and Preprocessing

Gene expression datasets of GC were systematically searched from the Cancer Genome Atlas (TCGA; https://cancergenome.nih.gov/) and the Gene Expression Omnibus (GEO; https://www.ncbi.nlm.nih.gov/geo/). In total, five GC datasets were gathered, namely, TCGA-STAD (*n* = 383), GSE84437 (*n* = 433) (Yoon et al., 2020), GSE62254 (*n* = 300) (Cristescu et al., 2015; Oh et al., 2018), GSE26901 (*n* = 109) (Oh et al., 2018), and GSE15459 (*n* = 192) (Lei et al., 2013). RNA-sequencing (RNA-seq) data of TCGA-STAD were retrieved from the Genomic Data Commons *via* TCGAbiolink package (Colaprico et al., 2016). RNA-seq data (FPKM value) were converted to TPM, resulting in a higher similarity to microarray data and higher compatibility between samples. The raw microarray data from the GEO repository were generated by Illumina and Affymetrix platforms. The raw data from Illumina were preprocessed with lumi package (Du et al., 2008). The raw data from Affymetrix were adjusted by background and standardized by quartile *via* Affy package (Gautier et al., 2004). Removal of batch effects was presented with ComBat function of sva package (Leek et al., 2012). Clinical features of all eligible GC datasets are summarized in Supplementary Table S1. The somatic mutation and copy number variation of TCGA-STAD were downloaded from the Genomic Data Commons. In total, 60 ferroptosis regulators were gathered from published research (Supplementary Table S2). RCircos package was implemented to visualize the genomic structure and positional relationships between ferroptosis regulators (Zhang et al., 2013). Somatic mutation in Mutation Annotation Format (MAF) was visualized through maftools package (Mayakonda et al., 2018).

### Functional Enrichment Analysis

Kyoto Encyclopedia of Genes and Genomes (KEGG) and Gene ontology (GO) enrichment analyses were carried out *via* clusterProfiler package (Yu et al., 2012). GO contained three categories: biological process, cellular component, and molecular function. The hallmark gene sets were acquired from the MSigDB database (http://software.broadinstitute.org/gsea/index.jsp) (Liberzon et al., 2015). Gene set variation analysis (GSVA) was carried out to infer the activation of hallmark pathways in GC specimens (Hänzelmann et al., 2013).

### Assessment of Drug Sensitivity

The Genomics of Drug Sensitivity in Cancer (GDSC) database (www.cancerRxgene.org) may provide drug sensitivity data from nearly 75,000 experiments that describe responses to 138 anticancer agents across nearly 700 cancer cells (Yang et al., 2013). Spearman correlation analysis was conducted to calculate the correlation between drug sensitivity and ferroptosis regulators.

### Tumor Microenvironment Immune-Infiltrating Analysis

Marker genes of infiltrating immune cells were gathered from the study by Bindea et al. (2013). The single sample gene set enrichment analysis (ssGSEA) method was utilized for quantifying the relative abundance of 28 immune cell types in GC specimens with GSVA package based on the reference transcriptomic data (Hänzelmann et al., 2013). The enrichment score was determined to indicate the relative abundance of each tumor-infiltrating immune cell.

### Unsupervised Clustering Analysis of Ferroptosis Regulators

Ferroptosis regulators were extracted from the five integrated datasets to identify distinct ferroptosis subtypes modulated by ferroptosis regulators. ConsensuClusterPlus package was implemented to estimate the number of unsupervised classes in the meta-cohort (Wilkerson and Hayes, 2010). The analysis was repeated 50 times to guarantee the reliability of clustering. The accuracy of clustering was verified *via* the t-distributed stochastic neighbor embedding (t-SNE) method according to the mRNA expression of ferroptosis regulators.

### Development of Ferroptosis Index

By adopting limma package, differential expression analysis was presented between three ferroptosis subtypes (Ritchie et al., 2015). Genes with adjusted *p* < 0.05 were retained, and shared differentially expressed genes (DEGs) were identified through a Venn diagram (Chen and Boutros, 2011). ConsensuClusterPlus package was applied for defining the number of genomic clustering based on the expression of shared DEGs. Univariate cox regression models were established to analyze the correlation between shared DEGs and GC prognosis. FPI was calculated *via* principal component analysis (PCA) based on the prognostic shared DEGs. Principal components (PC) 1 and 2 were extracted for defining FPI in line with the following formula: FPI = ∑(PC1i + PC2i), where i represented prognostic shared DEGs.

### Collection of Known Biological Signatures

This study gathered the gene sets of known biological signatures containing CD8 + T effector, DNA damage repair, pan-fibroblast TGF-β response signature (pan-F-TBRS), antigen-processing machinery, immune checkpoint, epithelial–mesenchymal transition (EMT1-3), FGFR3-related genes, angiogenesis, Fanconi anemia, cell cycle, DNA replication, nucleotide excision repair, homologous recombination, mismatch repair, WNT targets, cell cycle regulators, IFN-γ signatures, APM signaling, base excision repair, microRNAs in cancer, oocyte meiosis, p53 signaling pathway, progesterone-mediated oocyte maturation, proteasome, pyrimidine metabolism, spliceosome, and viral carcinogenesis (Rosenberg et al., 2016; Şenbabaoğlu et al., 2016; Mariathasan et al., 2018). Correlation between FPI score and these biological pathways was analyzed using the Spearman correlation test.

### Evaluation of Immune Response

Response to immune checkpoint blockade (ICB) was evaluated *via* the tumor immune dysfunction and exclusion (TIDE) computational method in line with the study by Hoshida et al. (Jiang et al., 2018). This method was based on two main mechanisms of tumor immune evasion: inducing T-cell dysfunction in tumors with high infiltration levels of cytotoxic T lymphocytes (CTLs) and preventing T-cell infiltration in tumors with low infiltration levels of CTLs.

### Collection of ICB Therapy Cohorts

This study gathered two ICB therapy cohorts: metastatic melanoma patients who received anti-PD-1 therapy from GSE78220 cohort (Hugo et al., 2016) and Liu et al. (2019). The mRNA expression data and follow-up information including survival time and therapeutic response [complete response (CR), partial response (PR), stable disease (SD), and progressive disease (PD)] were retrieved.

### Sensitivity to Chemotherapeutic Agents

The sensitivity to two chemotherapeutic agents (docetaxel and cisplatin) was predicted in each GC specimen. The half-maximal inhibitory concentration (IC50) was determined utilizing ridge regression analysis through pRRophetic package (Geeleher et al., 2014).

### Prediction of Potential Small-Molecular Compounds

This study retrieved drug sensitivity data of human cancer cell lines (CCLs) from the CTRP (https://portals.broadinstitute.org/ctrp) and PRISM (https://depmap.org/portal/prism/) projects. The area under the curves (AUCs) were utilized for evaluating response to each drug. The AUC value was inversely proportional to drug sensitivity. The mRNA expression profiling in the Cancer Cell Line Encyclopedia (CCLE) database (https://portals.broadinstitute.org/ccle/) was employed for CTRP and PRISM analyses (Ghandi et al., 2019).

### Statistical Analyses

All statistical analyses were conducted with R (https://www.r-project.org/). Univariate Cox regression analysis was applied to assess the correlation between overall survival and ferroptosis regulators in GC patients with survival package. The Kaplan–Meier survival analysis was carried out utilizing survival and survminer packages. The Spearman correlation test was utilized for inferring the correlation between two parameters. Student’s t and Wilcoxon tests were adopted to compare the differences between two groups, while one-way ANOVA and Kruskal–Wallis tests were conducted to compare the differences between three or more groups. Two-sided *p*-values < 0.05 were considered statistically significant.

## Results

### Landscape of Expression, Genetic Mutation, and DNA Methylation of Ferroptosis Regulators in Gastric Cancer

**FIGURE 1:**
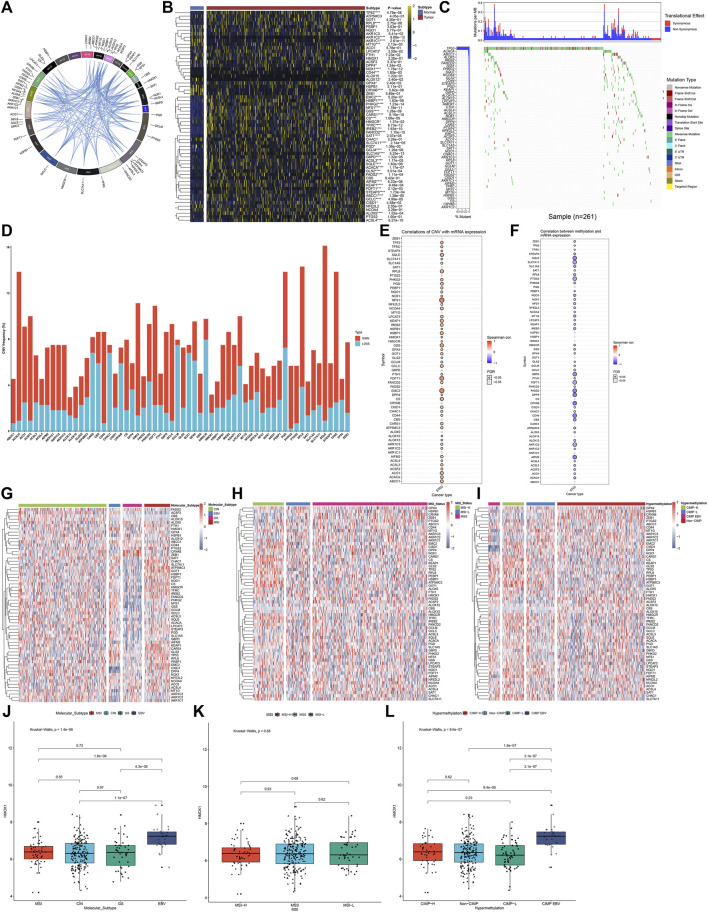
Landscape of expression, genetic mutation, and DNA methylation of ferroptosis regulators in GC. **(A)** RCircos plots showing the genomic location and relationships among 60 ferroptosis regulators. **(B)** Heatmap visualizing the mRNA expression of ferroptosis regulators in 32 normal and 375 GC tissues in the TCGA-STAD dataset. Yellow, upregulation; blue, downregulation. *p < 0.05; ***p < 0.001; ****p < 0.0001. **(C)** Waterfall diagram for the genetic mutation of ferroptosis regulators across 261 GC specimens. The left indicated the mutation rate of each ferroptosis regulator. Each column represented each sample. Each mutation type was identified by a unique color. The upper indicated mutations per MB in each ferroptosis regulator. **(D)** Stacked diagram visualizing the frequency of CNV across GC specimens. Red, amplification; blue, deletion. **(E)** Spearman correlation between mRNA expression and CNV in ferroptosis regulators across GC samples. Red, positive correlation; blue, negative correlation. The size of the bubble was proportional to the correlation coefficient. **(F)** Spearman correlation between mRNA expression and DNA methylation in ferroptosis regulators across GC samples. Red, positive correlation; blue, negative correlation. The size of the bubble was proportional to the correlation coefficient. **(G)** Heatmap showing the mRNA expression of ferroptosis regulators among four molecular subtypes: CIN, EBV, GS, and MSI. **(H)** Heatmap visualizing the expression distribution of ferroptosis regulators among three MSI status-based subtypes: MSI-H, MSI-L, and MSS. **(I)** Heatmap for the mRNA expression of ferroptosis regulators among four DNA methylation-based subtypes: CIMP-H, CIMP-L, CIMP-EBV, and non-CIMP. **(J–L)** Distribution of the mRNA expression of HMOX1 across different subtypes, molecular subtypes, MSI status-based subtypes, and DNA methylation-based subtypes.

This study gathered 60 ferroptosis regulators and their roles were observed in GC. Figure 1A visualized the genomic position and relationships between ferroptosis regulators. First, we analyzed the mRNA expressional alterations of ferroptosis regulators in 32 normal and 375 GC tissues in the TCGA-STAD dataset. There was the expressional imbalance of ferroptosis regulators and most presented the distinct upregulation in GC compared to normal tissues (Figure 1B). We further ascertained whether the genetic variation affected the expression of ferroptosis regulators in GC. Among 261 GC specimens, we observed that TP53 displayed the highest mutation rate, followed by ACACA and ABCC1 (Figure 1C). Further analysis showed that CNV widely occurred in ferroptosis regulators and amplification was the major mutation type (Figure 1D). The Spearman correlation analysis also confirmed that amplification of CNV exhibited a positive correlation to mRNA expression in ferroptosis regulators across GC (Figure 1E). DNA methylation acts as the main epigenetic modification, which transcriptionally regulates gene expression. Herein, we observed that DNA methylation had negative association to mRNA expression in ferroptosis regulators (Figure 1F). Collectively, our findings were indicative that amplification of CNV and hypermethylation might prominently result in the overexpression of ferroptosis regulators in GC. The TCGA project has revealed four molecular subtypes of GC: Epstein–Barr virus (EBV), microsatellite instability (MSI), genomically stable (GS), and chromosomal instability (CIN) (Sohn et al., 2017). Among the four molecular subtypes, the expression of ferroptosis regulators showed wide heterogeneity (Figure 1G). Most ferroptosis regulators were upregulated in MSI but were downregulated in GS. According to MSI status, GS can be divided into MSI-high (MSI-H), MSI-low (MSI-L), and MSS. We observed that MSI-H tumors displayed the significant up-regulation of most ferroptosis regulators (Figure 1H). Moreover, we compared the expression of ferroptosis regulators in four DNA methylation-based subtypes: CIMP-high (CIMP-H), CIMP-low (CIMP-L), CIMP-EBV, and non-CIMP. As shown in Figure 1I, ferroptosis regulators exhibited relatively high expression in CIMP-L. We visualized the expression of a ferroptosis regulator (HMOX1) across different molecular subtypes (Figures 1J–L). The results showed that HMOX1 exhibited the highest expression in EBV and CIMP-EBV but did not have significant difference among different MSI subtypes.

### Biological Function, Prognostic Implication, and Immune Cell Infiltration Correlation of Ferroptosis Regulators in Gastric Cancer

**FIGURE 2:**
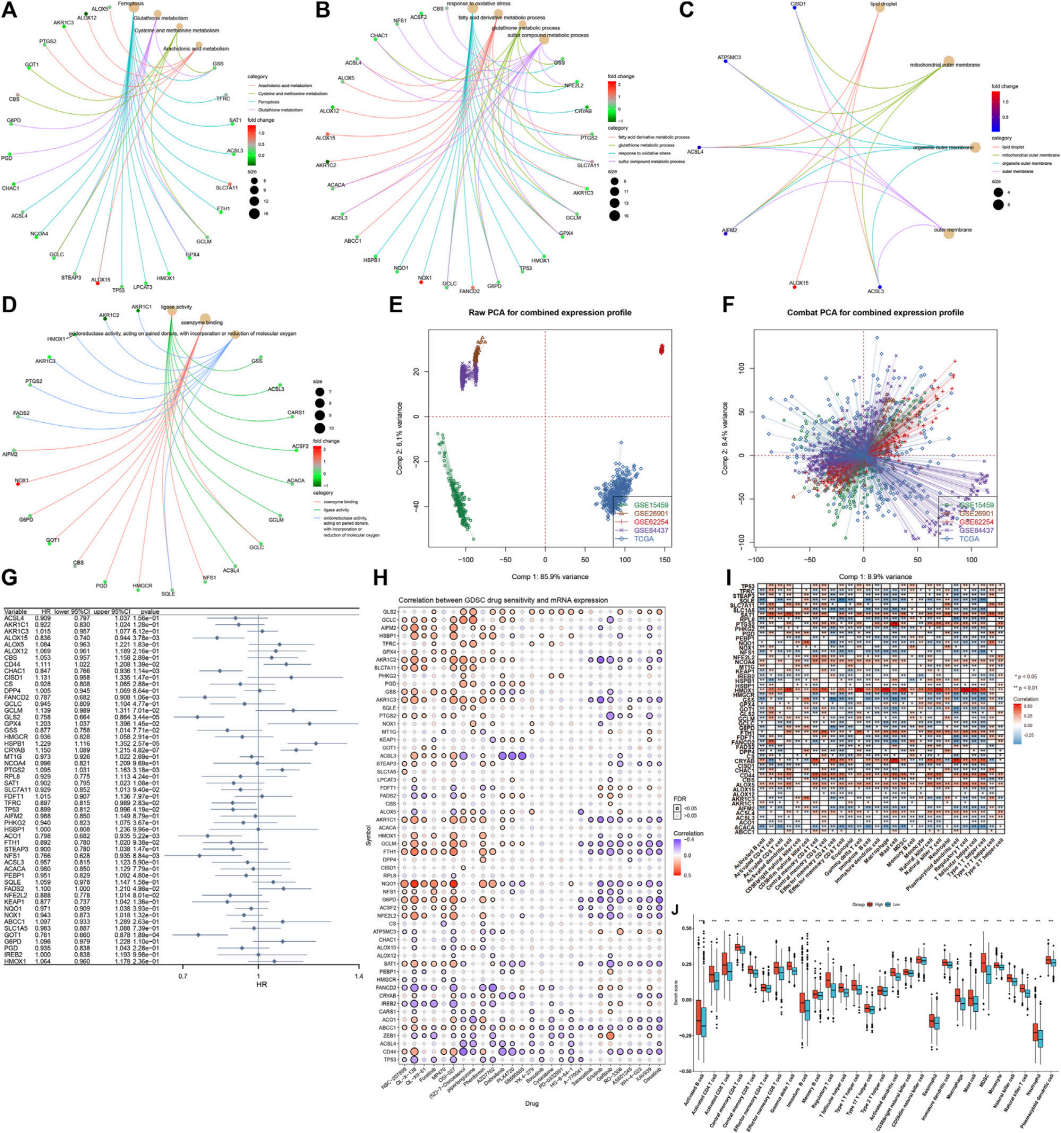
Biological function, prognostic implication, and immune cell infiltration correlation of ferroptosis regulators in the GC meta-cohort. **(A)** KEGG pathways involved in ferroptosis regulators. **(B–D)** Biological processes, cellular components, and molecular functions enriched by ferroptosis regulators. **(E)** Integration of the mRNA expression data of five GC datasets: TCGA-STAD, GSE84437, GSE62254, GSE26901, and GSE15459. **(F)** Removal of batch effects. **(G)** Forest plot showing the association between ferroptosis regulators and GC prognosis through univariate Cox regression models. **(H)** Spearman correlation analysis between ferroptosis regulators and drug sensitivity *via* the GDSC database. Red, positive correlation; blue, negative correlation. The size of the bubble was proportional to the correlation coefficient. **(I)** Spearman correlation analysis between ferroptosis regulators and tumor-infiltrating immune cells by ssGSEA method. Red, positive correlation; blue, negative correlation. **(J)** Comparisons of enrichment scores of tumor-infiltrating immune cells in high and low HMOX1 expression GC samples. *p < 0.05; **p < 0.01; and ***p < 0.001.

Through clusterProfiler package, we evaluated the pathways involved in ferroptosis regulators. In Figure 2A, arachidonic acid metabolism, cysteine and methionine metabolism, and ferroptosis and glutathione metabolism were mainly enriched by ferroptosis regulators. GO enrichment analysis uncovered that ferroptosis regulators markedly participated in the fatty acid derivative metabolic process, glutathione metabolic process, response to oxidative stress, and sulfur compound metabolic process (Figure 2B). Cellular components including lipid droplet, mitochondrial outer membrane, organelle outer membrane, and outer membrane were primarily regulated by ferroptosis regulators (Figure 2C). In Figure 2D, ferroptosis regulators possessed the molecular functions of coenzyme binding, ligase activity, oxidoreductase activity, and acting on paired donors, with incorporation or reduction of molecular oxygen. Aforementioned data were indicative of the critical biological function of ferroptosis regulators in the occurrence and progression of GC. This study gathered and merged five GC datasets: TCGA-STAD, GSE84437, GSE62254, GSE26901, and GSE15459 (Figure 2E). Batch effects were corrected *via* ComBat function of sva package (Figure 2F). In the meta-cohort, univariate Cox regression analysis uncovered that 15 ferroptosis regulators, namely, CRYAB, HSPB1, GLS2, GOT1, FANCD2, CHAC1, PTGS2, ALOX15, ACO1, NFS1, CD44, GPX4, TFRC, TP53, and FADS2 exhibited significant correlations to GC prognosis (Figure 2G; Supplementary Figure S1). Drug sensitivity was also estimated in GC specimens *via* the GDSC database. As shown in the Spearman correlation analysis, ferroptosis regulators were significantly correlated to the sensitivity to NSC-207895, QL-X-138, QL-XII-61, foretinib, MP470, OSI-027, (5Z)-7-oxozeaenol, piperlongumine, phenformin, AZD7762, dabrafenib, PLX4720, SB590885, YK 4-279, bosutinib, cytarabine, PD-0332991, HG-6-64-1, A-770041, saracatinib, erlotinib, gefitinib, RO-3306, AS601245, WH-4-023, XAV939, and dasatinib across GC samples (Figure 2H). Using the ssGSEA method, we inferred the enrichment score of 28 immune cells. As depicted in Figure 2I, ferroptosis regulators displayed distinct associations with TME immune infiltration, indicative of the interactions between ferroptosis and tumor immunity in GC. Among them, high HMOX1 expression was in relation to increased infiltration levels of tumor-infiltrating immune cells across GC (Figure 2J).

### Establishment of Three Ferroptosis Regulator-Mediated Subtypes With Different Prognosis and Tumor Microenvironment Immune Infiltration

**FIGURE 3:**
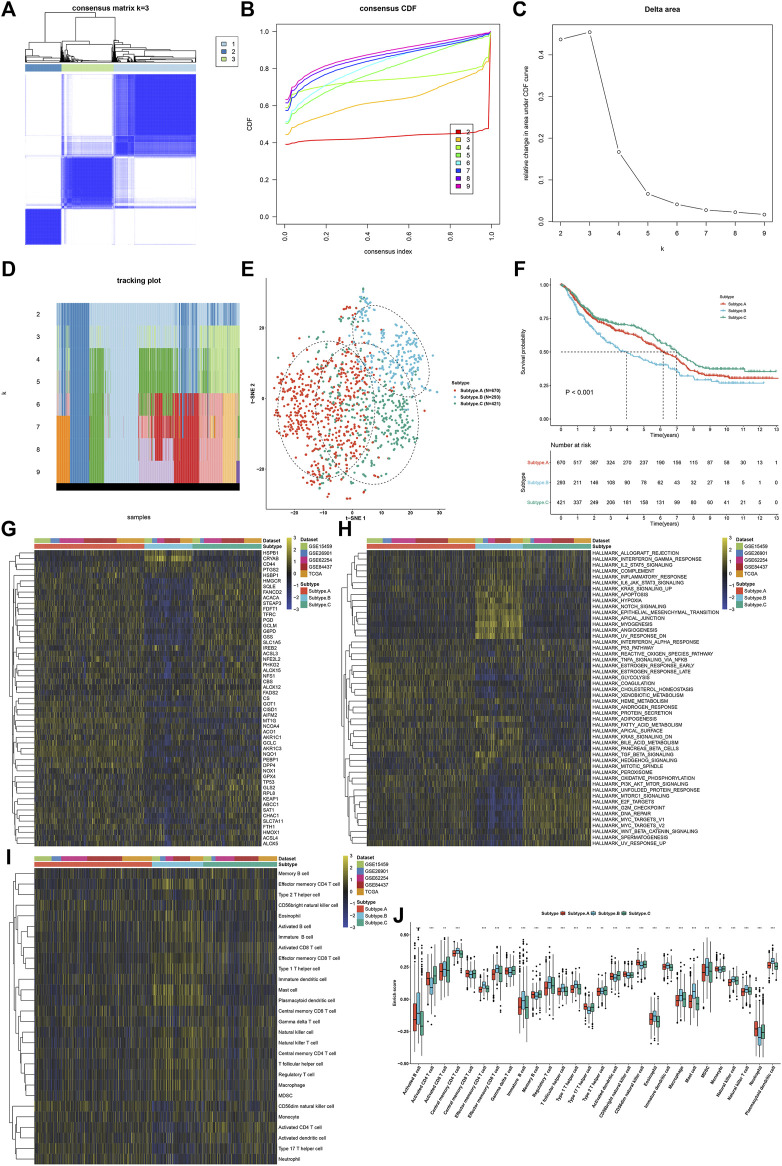
Establishment of three ferroptosis regulator-mediated subtypes with different prognosis and TME immune infiltration in the GC meta-cohort. **(A)** Consensus matrix when k = 3. **(B)** Empirical CDF plots displaying the consensus distribution corresponding to each k. **(C)** Delta area diagram visualizing the relative change in area under CDF curve at different k. **(D)** Item tracking plot showing the consensus clustering of items (in column) at each k value (in row). **(E)** The t-SNE plots verifying the difference among three ferroptosis subtypes according to the mRNA expression of ferroptosis regulators. **(F)** Survival analysis for GC patients in three ferroptosis subtypes. **(G)** Heatmap showing the mRNA expression of ferroptosis regulators in three ferroptosis subtypes. Yellow, up-regulation; blue, down-regulation. **(H)** Heatmap for the activity of the 50 hallmark pathways in three ferroptosis subtypes. Yellow, up-regulation; blue, down-regulation. **(I)** Heatmap visualizing the infiltration levels of tumor-infiltrating immune cells in three ferroptosis subtypes. **(J)** Comparison of the infiltration levels of tumor-infiltrating immune cells in three ferroptosis subtypes. **p < 0.01; ***p < 0.001.

This study applied the ConsensusClusterPlus computational method to classify GC patients in the meta-cohort into three ferroptosis subtypes according to the expression profiling of 60 ferroptosis regulators (Figure 3A). Cumulative distribution function (CDF) was employed to identify the k value at which the distribution reached an approximate maximum that indicated a maximum stability (Figure 3B). Delta area plot showed that when k = 3, the area under the curve only slightly increased (Figure 3C). Furthermore, the item tracking plot showed the consensus clustering of items at different k values (Figure 3D). Collectively, GC patients were clustered into three ferroptosis subtypes with high stability, namely, as ferroptosis subtype A, B, and C. The t-SNE plots confirmed the difference among three ferroptosis subtypes according to the mRNA expression of ferroptosis regulators (Figure 3E). Survival analysis uncovered that ferroptosis subtype B possessed the worst survival outcomes, while ferroptosis subtype C had the significant survival advantage (Figure 3F). In Figure 3G, we observed that most ferroptosis regulators were distinctly downregulated in ferroptosis subtype B. The activity of hallmark pathways was estimated *via* the ssGSEA computational method. Carcinogenic pathways including KRAS signaling, hypoxia, Notch signaling, and hedgehog signaling were markedly activated in ferroptosis subtype B, indicative of the poor prognosis (Figure 3H). Moreover, stromal activation pathways including epithelial–mesenchymal transition, angiogenesis, and TGF-β signaling, as well as immune activation pathways including allograft rejection, interferon *γ* response, IL2-STAT5 signaling, complement, inflammatory response, and IL6-JAK-STAT3 signaling displayed significant upregulation in ferroptosis subtype B. Tumor-infiltrating immune cells were then quantified. In Figures 3I,J, we observed that ferroptosis subtype B displayed high infiltration levels of innate immune cells (such as natural killer cells, macrophages, eosinophils, mast cells, MDSCs, and plasmacytoid dendritic cells) and adaptive immune cells (such as effector memory CD4^+^ T cells, activated B cells, activated CD8^+^ T cells, effector memory CD8^+^ T cells, central memory CD8^+^ T cells, and central memory CD4^+^ T cells).

### Identification of Three Ferroptosis Genomic Subtypes Characterized by Different Prognosis and Tumor Microenvironment Features

**FIGURE 4:**
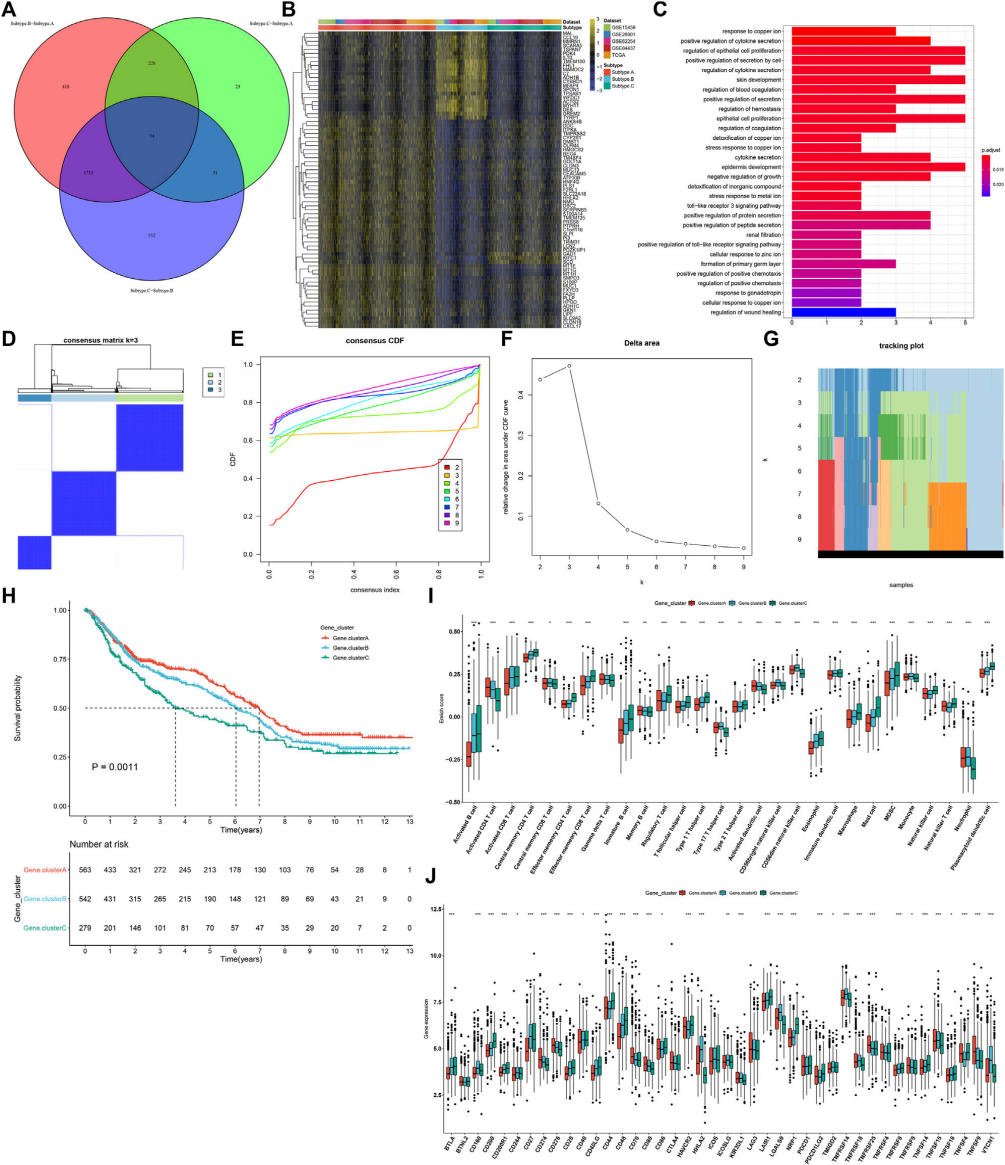
Identification of three ferroptosis genomic subtypes characterized by different prognosis and TME features in the GC meta-cohort. **(A)** Shared DEGs between three ferroptosis subtypes. **(B)** Heatmap showing the mRNA expression of the shared DEGs in each ferroptosis subtype. Yellow, up-regulation; blue, down-regulation. **(C)** GO annotation results of the shared DEGs. **(D)** Consensus matrix when k = 3. **(E)** Empirical CDF plots displaying the consensus distribution corresponding to each k. **(F)** Delta area diagram visualizing the relative change in area under CDF curve at different k. **(G)** Item tracking plot showing the consensus clustering of items (in column) at each k value (in row). **(H)** Survival analysis of GC patients in three ferroptosis genomic subtypes. **(I)** Comparison of the relative abundance of tumor-infiltrating immune cells among three ferroptosis genomic subtypes. **(J)** Comparison of the mRNA expression of immune checkpoints among three ferroptosis genomic subtypes. *p < 0.05; **p < 0.01; ***p < 0.001.

In total, 74 shared DEGs were identified among three ferroptosis subtypes (Figure 4A; Supplementary Table S3). Figure 4B depicts the difference in mRNA expression of these shared DEGs between ferroptosis subtypes. GO annotation analysis uncovered that these shared DEGs were distinctly involved in mediating stromal activation-related processes (like regulation of epithelial cell and epithelial cell proliferation) and immune-relevant processes (like positive regulation of cytokine secretion, regulation of cytokine secretion, and cytokine secretion; Figure 4C). By unsupervised clustering analysis, we established three ferroptosis genomic subtypes across GC patients in the meta-cohort, namely, ferroptosis genomic subtype A–C (Figures 4D–G). In Figure 4H, ferroptosis genomic subtype C exhibited the poorest prognosis, whereas ferroptosis genomic subtype A possessed the most favorable prognosis. Furthermore, we observed that ferroptosis genomic subtype C had the high infiltration levels of innate immune cells (like natural killer cells, macrophages, eosinophils, mast cells, MDSCs, and plasmacytoid dendritic cells; Figure 4I). In Figure 4J, ferroptosis genomic subtype C had the highest expression of immune checkpoints BTLA, CD200, CD200R1, CD28, CD40LG, CD44, CD48, LAIR1, NRP1, PDCD1LG2, TNFRSF14, TNFRSF18, and TNFRSF8; ferroptosis genomic subtype B exhibited the highest expression of CD160, CD27, HHLA2, ICOSLG, KIR3DL1, LGALS9, TMIGD2, TNFSF14, and VTCN1; ferroptosis genomic subtype A displayed the highest expression of CD274, CD276, CD70, CD80, TNFRSF25, TNFRSF9, and TNFSF9.

### Development of Ferroptosis Index Score and Evaluation of Its Relevant Clinical Features

**FIGURE 5:**
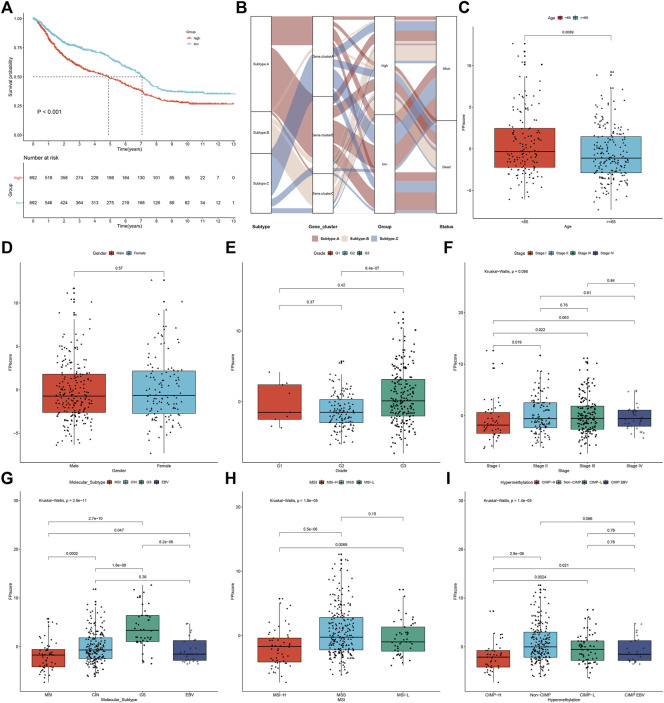
Development of FPI score and evaluation of its relevant clinical features in the meta-cohort. **(A)** Survival analysis of GC patients in high and low FPI groups. **(B)** Sankey diagram showing the interactions among ferroptosis subtypes, ferroptosis genomic subtypes, FPI, and survival status. **(C–F)** Comparison of FPI score in different clinical features, including age (age<65 years vs. age≥65 years), gender (female vs. male), grade (G1 vs. G2 vs. G3), and stage (stage I vs. stage II vs. stage III vs. stage IV). **(G–I)** Comparison of the FPI score in different subtypes, including molecular subtypes (MSI, CIN, GS, and EBV), MSI status-based subtypes (MSI-H, MSS, and MSI-L), and DNA methylation-based subtypes (CIMP-H, CIMP-L, CIMP-EBV, and non-CIMP).

As shown in univariate Cox regression models, 22 shared DEGs were in relation to GC prognosis (Table 1). Using the PCA computational method, we developed an FPI score to quantify ferroptosis subtypes following the expression of the prognosis-related shared DEGs. High FPI was indicative of unfavorable clinical outcomes in comparison to the low FPI score (Figure 5A). Sankey diagram visualized the interactions among ferroptosis subtypes, ferroptosis genomic subtypes, FPI, and survival status (Figure 5B). The clinical implications of FPI were evaluated in depth. In Figure 5C, patients aged <65 years had a higher FPI score than those aged ≥65 years. No significant difference was investigated between female and male patients (Figure 5D). For grade, G3 exhibited the highest FPI score (Figure 5E). Stages II–IV had higher FPI scores than stage I (Figure 5F). These indicated that FPI score might predict the severity of GC. Among four molecular subtypes, GS subtype displayed the highest FPI score (Figure 5G). For MSI status, MSS subtype exhibited the highest FPI score (Figure 5H). Furthermore, among DNA methylation-based subtypes, non-CIMP had the highest FPI score (Figure 5I).

**TABLE 1:** Identification of 22 prognosis-related shared DEGs in GC through univariate Cox regression models.

Shared DEGs	HR	Low 95% CI	High 95% CI	*P*
FHL1	1.092087	1.041977	1.144606	0.000237
MYH11	1.07675	1.037586	1.117393	9.16E-05
DES	1.071609	1.038122	1.106175	1.96E-05
C7	1.085066	1.041192	1.130789	0.000106
MFAP4	1.094248	1.040893	1.150339	0.000413
MAMDC2	1.090156	1.031244	1.152433	0.002324
ACTG2	1.091419	1.048324	1.136287	2.08E-05
SPON1	1.116166	1.056533	1.179165	8.74E-05
TMEM125	0.918597	0.854042	0.988033	0.022384
ITPKA	0.908395	0.854178	0.966052	0.002214
SMPD3	0.819972	0.753313	0.892528	4.47E-06
HNF4G	0.911258	0.851557	0.975145	0.007188
TMPRSS2	0.941752	0.890433	0.996028	0.035801
CYBRD1	1.148231	1.074373	1.227167	4.61E-05
IL33	1.099016	1.033994	1.168126	0.002411
MMRN1	1.110902	1.034905	1.19248	0.003627
DDC	0.952129	0.907629	0.998811	0.04457
PDK4	1.123587	1.064525	1.185927	2.34E-05
WFDC1	1.078939	1.001167	1.162753	0.046534
LIPF	0.966674	0.941203	0.992834	0.012851
TYRP1	1.078591	1.013015	1.148412	0.018078
TPSAB1	1.083159	1.026209	1.14327	0.003746

### Association Between Ferroptosis Index Score and Tumor Immunity

**FIGURE 6:**
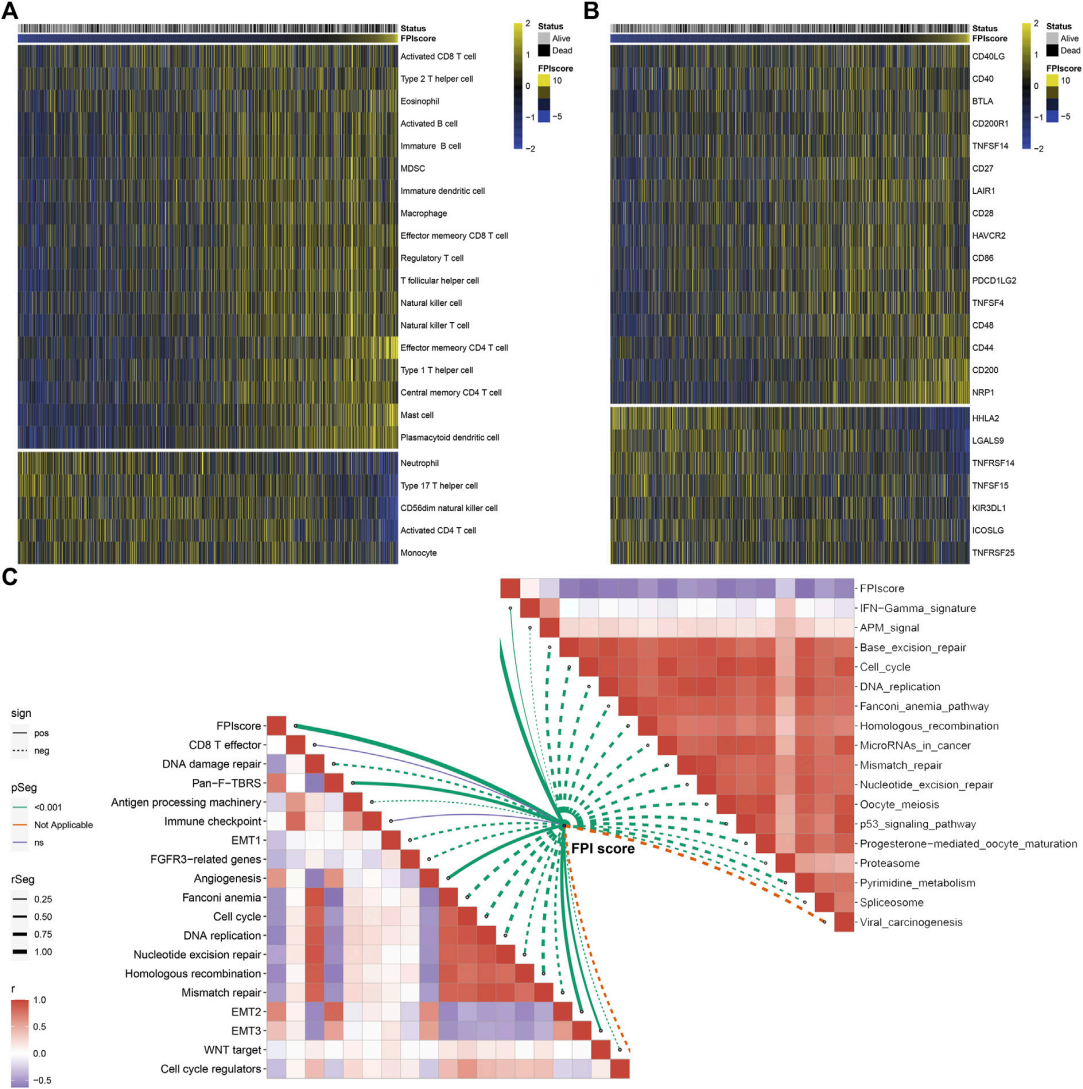
Association between FPI score and tumor immunity across GC samples. **(A)** Heatmap visualizing the infiltration levels of tumor-infiltrating immune cells in low and high FPI GC samples. Yellow, high infiltration; blue, low infiltration. **(B)** Heatmap showing the mRNA expression of immune checkpoints in low and high FPI GC samples. Yellow, high expression; blue, low expression. **(C)** Association between FPI score and known biological processes and pathways.

Further analysis uncovered that high FPI samples exhibited high infiltration levels of innate immune cells (such as natural killer cells, macrophages, eosinophils, mast cells, MDSCs, and plasmacytoid dendritic cells) and adaptive immune cells (such as effector memory CD4^+^ T cells, activated B cells, activated CD8^+^ T cells, effector memory CD8^+^ T cells, central memory CD8^+^ T cells, and central memory CD4^+^ T cells; Figure 6A). Most immune checkpoints had increased mRNA expression in patients with a high FPI score including CD40LG, CD40, BTLA, CD200R1, TNFSF14, CD27, LAIR1, CD28, HAVCR2, CD86, PDCD1LG2, TNFSF4, CD48, CD44, CD200, and NRP1 (Figure 6B). Meanwhile, HHLA2, LGALS9, TNFRSF14, TNFSF15, KIR3DL1, ICOSLG, and TNFRSF25 were markedly upregulated in low FPI samples. The FPI score was positively correlated to stromal activation pathways including pan-F-TBRS, angiogenesis, and EMT (Figure 6C). Moreover, we observed that the FPI score exhibited negative correlations to DNA damage repair, cell cycle, DNA replication, nucleotide excision repair, homologous recombination, and mismatch repair as well as carcinogenic pathways such as p53 signaling pathway, mismatch in cancer, and viral carcinogenesis.

### Ferroptosis Index Score Serves as a Predictor of Immune Response

**FIGURE 7:**
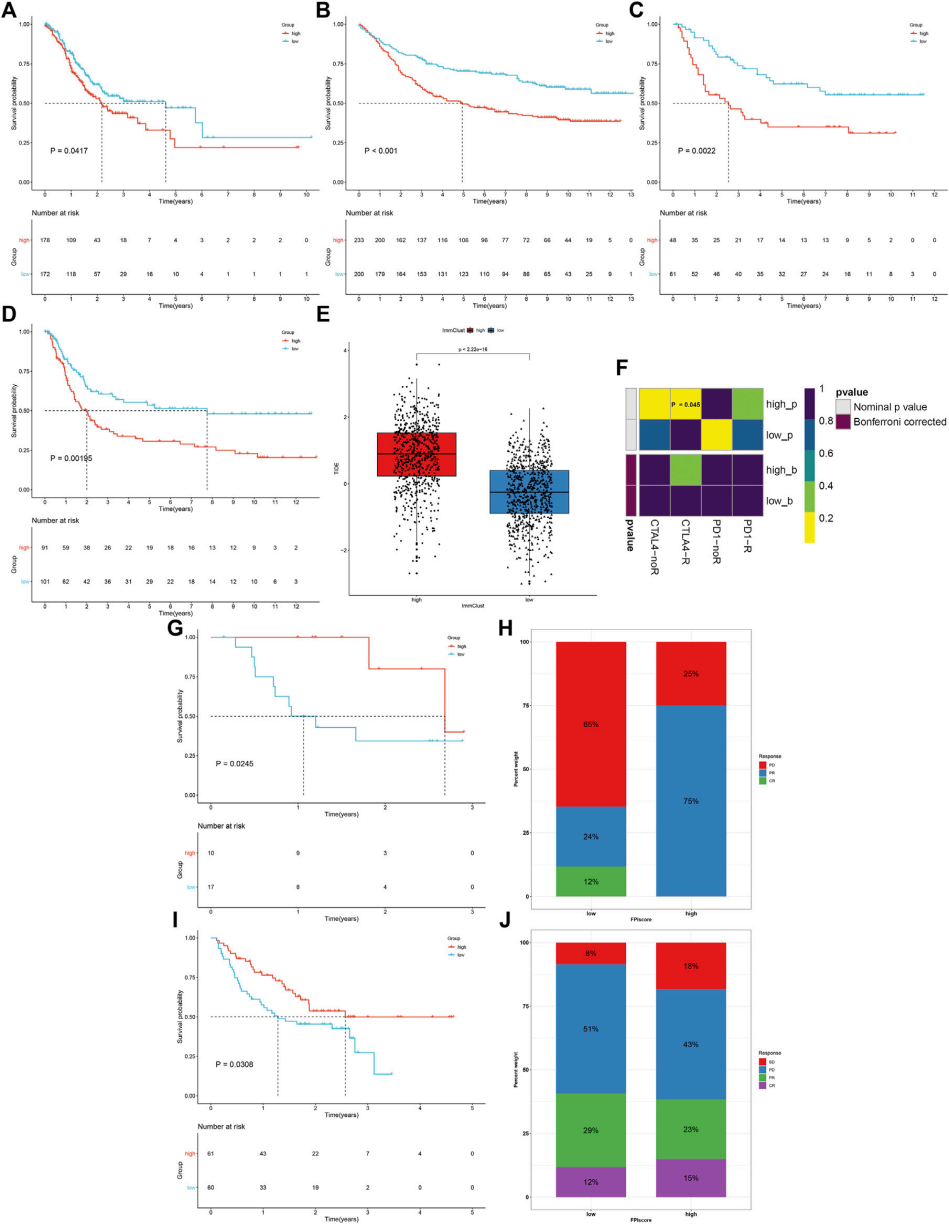
FPI score serves as a predictor of immune response. **(A–D)** Survival analysis of GC patients with high and low FPI scores in the TCGA-STAD, GSE84437, GSE26901, and GSE15459 cohorts. **(E)** Comparison of the TIDE score in high and low FPI score groups. **(F)** SubMap analysis estimating the response to anti-CTLA-4 and anti-PD-1 therapy for high and low FPI patients. **(G,H)** Comparison of survival outcomes and therapeutic response to anti-PD-1 therapy between high and low FPI patients in the GSE78220 cohort. **(I,J)** Comparison of survival outcomes and therapeutic response to anti-PD-1 therapy between high and low FPI patients in the Liu et al. cohort.

Prognostic implications of the FPI score were validated in the TCGA-STAD, GSE84437, GSE26901, and GSE15459 cohorts. Consistently, high FPI score was indicative of more unfavorable survival outcomes than low FPI score in each GC cohort (Figures 7A–D). The TIDE score was determined in each HCC specimen. In comparison to high FPI, a reduced TIDE score was observed in low FPI (Figure 7E), which indicated that low FPI patients were more likely to benefit from immunotherapy. SubMap analysis showed that patients with a high FPI score were more likely to respond to anti-CTLA-4 therapy (Figure 7F). Two anti-PD-1 therapy cohorts were gathered. In the GSE78220 cohort, patients with high FPI exhibited the distinct survival advantage (Figure 7G). Patients with high FPI had higher therapeutic response than those with low FPI (75% vs. 36%; Figure 7H). Similar results were found in the Liu et al. cohort (Figures 7I,J).

### Prediction of Potential Therapeutic Agents Against Gastric Cancer Based on the Ferroptosis Index Score

**FIGURE 8:**
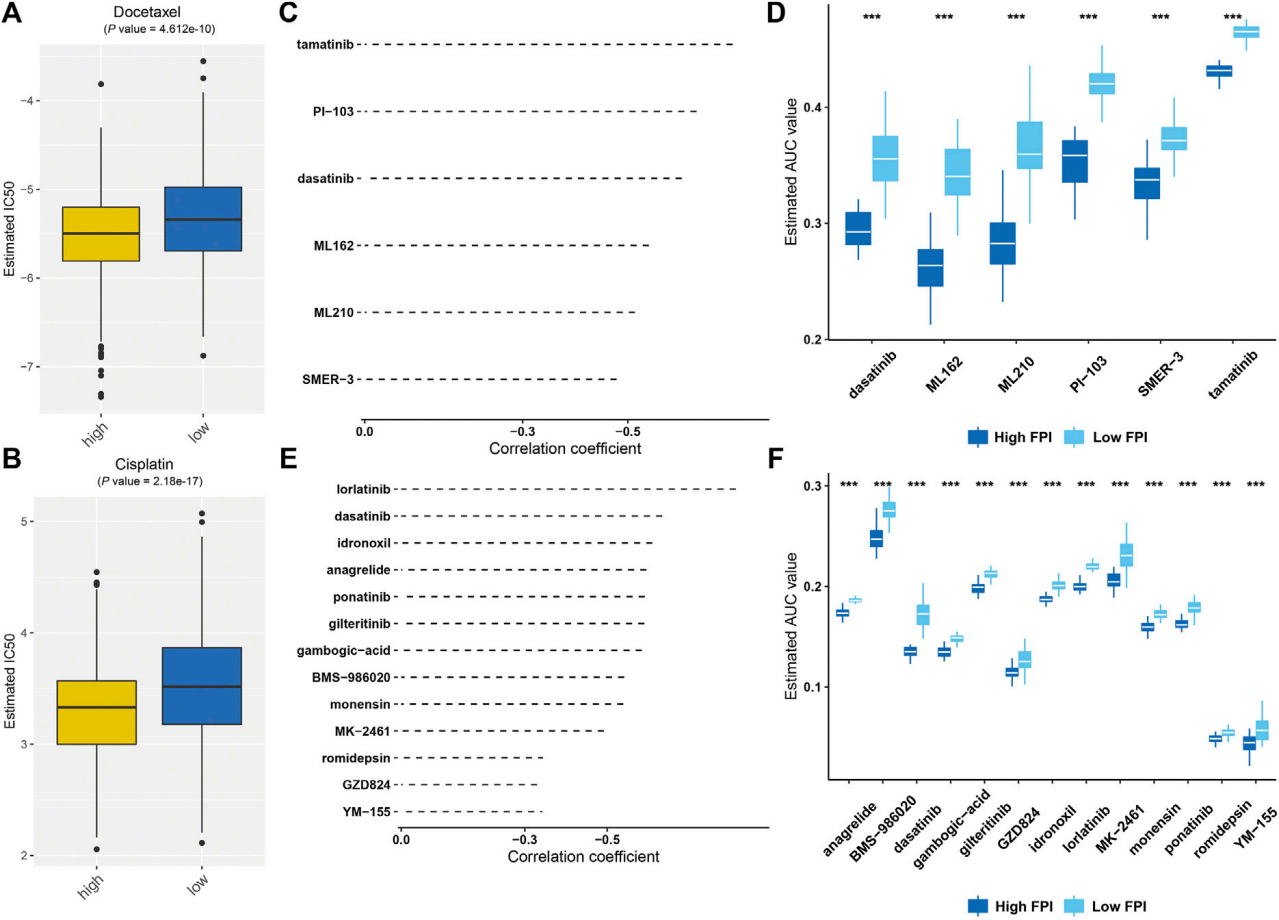
Prediction of potential therapeutic agents against GC based on FPI score. **(A,B)** Comparison of estimated IC50 values of docetaxel and cisplatin in high and low FPI score patients. **(C)** Spearman correlation analysis between FPI score and six CTRP-derived compounds. **(D)** Comparison of the estimated AUC value of six CTRP-derived compounds in high and low FPI score patients. **(E)** Spearman correlation analysis between FPI score and thirteen PRISM-derived compounds. **(F)** Comparison of estimated AUC value of thirteen PRISM-derived compounds in high and low FPI score patients. ***p < 0.001.

We further estimated response to chemotherapy agents, docetaxel and cisplatin. As a result, high FPI samples displayed significantly reduced IC50 values of docetaxel and cisplatin compared with low FPI samples (Figures 8A,B), suggesting that high FPI was indicative of higher sensitivity to docetaxel and cisplatin. Furthermore, we predicted six CTRP-derived compounds based on the FPI score, including ML162 (*r* = −0.54), PI-103 (*r* = −0.63), tamatinib (*r* = −0.70), dasatinib (*r* = −0.60), ML210 (*r* = −0.51), and SMER-3 (*r* = −0.48; Figure 8C). As shown in Figure 8D, patients with a high FPI score were more likely to respond to ML162, PI-103, tamatinib, dasatinib, ML210, and SMER-3. Meanwhile, thirteen PRISM-derived compounds were also predicted, containing monensin (*r* = −0.54), gambogic-acid (*r* = −0.58), BMS-986020 (*r* = −0.54), GZD824 (*r* = −0.36), ponatinib (*r* = −0.59), dasatinib (*r* = −0.63), MK-2461 (*r* = −0.49), romidepsin (*r* = −0.38), anagrelide (*r* = −0.59), gilteritinib (*r* = −0.59), idronoxil (−0.61), YM-155 (−0.35), and lorlatinib (*r* = −0.81; Figure 8E). Patients with a high FPI score were more likely to benefit from aforementioned compounds (Figure 8F).

## Discussion

Ferroptosis is a type of iron-dependent necrosis (Chen et al., 2021), which displays morphological, biochemical, and genetic difference from other types of cell death (Friedmann Angeli et al., 2019). Emerging evidence suggests the pivotal roles of ferroptosis in GC pathogenesis (Lee et al., 2020). However, most studies focus on a single ferroptosis regulator, and the overall ferroptosis characterization mediated by a variety of ferroptosis regulators remains not comprehensively determined. Identification of the roles of different ferroptosis-based subtypes may contribute to improving our understanding of ferroptosis in forming TME diversity and complexity, and guiding individualized treatment. Hence, our findings provided a systematic analysis of ferroptosis regulators and characterized the implications of ferroptosis in GC.

Five GC cohorts, namely, TCGA-STAD, GSE84437, GSE62254, GSE26901, and GSE15459, were integrated in this study. We collected 60 ferroptosis regulators and most regulators were distinctly upregulated in GC, indicating the activation of ferroptosis in GC. We investigated that the aberrant expression of ferroptosis regulators was mainly modulated by copy number variation and methylation in GC. Most ferroptosis regulators were in relation to GC prognosis, drug sensitivity, and tumor-infiltrating immune cells, revealing their critical roles in GC progression. Based on them, three ferroptosis subtypes were conducted, characterized by different survival outcomes, hallmark pathway activity, and TME features across GC. The establishment of GC ferroptosis subtypes could accurately predict the patients’ clinical outcomes, and TME status. To quantify the ferroptosis level, we developed FPI based on the expression of prognostic ferroptosis-relevant genes. High FPI was indicative of unfavorable clinical outcomes as well as increased grade and stage in GC subjects. This indicated that FPI acted as a reliable tool to assist clinicians in the prediction of GC prognosis as well as to facilitate personalized therapy. GC has pathological and molecular heterogeneity (Qiu et al., 2020). Thus, it is of significance to develop stable prognostic indicators. Our findings indicated that FPI could be applied to comprehensively assess the ferroptosis-based subtypes and their corresponding TME characterization within individual GC patients, and thus guided more effective treatment strategies.

High FPI was in relation to high infiltration levels of innate and adaptive immune cells. Immunotherapy based on ICB has achieved considerable progress; nevertheless, only one-third of patients benefit from ICB (Tang et al., 2020). Ferroptosis induction combined with ICB exhibits synergistically strengthened antitumor activity (Wang et al., 2019). Our results uncovered that FPI might be utilized for predicting response to anti-CTLA4 and anti-PD-1 therapy. This indicated that FPI acted as a promising predictor of immune response. Cisplatin and docetaxel are common chemotherapeutic agents for GC. However, advanced GC patients often acquire resistance to chemotherapy, leading to the median overall survival of only 8–11 months (Zhao et al., 2018). Experimental evidence demonstrates that inducing ferroptosis can overcome resistance to chemotherapy. For instance, ferroptosis induction may alleviate cisplatin resistance in GC through restraining the Nrf2/Keap1/xCT pathway (Fu et al., 2021). SIRT6 silencing may overcome resistance to sorafenib through activating ferroptosis in GC (Cai et al., 2021). Ferroptosis induced by erastin may reverse ABCB1-mediated docetaxel resistance in ovarian cancer (Zhou et al., 2019). Here, high FPI was in relation to the increased sensitivity to cisplatin and docetaxel in GC. Also, six CTRP-derived compounds and thirteen PRISM-derived compounds were predicted for GC patients with high FPI. Hence, the FPI score might be utilized for predicting the therapeutic response of GC patients to chemotherapy. Nevertheless, more experiments are required to verify the clinical efficacy of above compounds against GC.

Nevertheless, our study has certain limitations. First, although ferroptosis-based GC subtypes were verified through multiple datasets and algorithms, more robust experiments are essential for gaining more insights into the underlying mechanisms of different ferroptosis subtypes. Second, independent external datasets and reliable approaches were employed for confirming the FPI score. However, the FPI score was computed and verified on the basis of retrospective data from publicly available datasets. Hence, large-scale prospective clinical cohorts are needed for evaluating its effectiveness and practicability.

## Conclusion

Taken together, this study carried out a systematic analysis of genomic alterations and expression profiling of ferroptosis regulators in GC. We established three ferroptosis subtypes characterized by different prognosis and tumor-infiltrating immune cells. Moreover, FPI was computed to evaluate the ferroptosis levels according to the expression of prognostic ferroptosis-related signatures. The FPI score was in relation to survival outcomes, hallmark pathways, TME, chemotherapy resistance, and immune response. Our findings highlighted the pivotal roles of ferroptosis as well as the potential of ferroptosis-related therapy in GC. Collectively, the FPI score enabled to comprehensively assess the ferroptosis-based subtypes and characterize TME features for each GC patient, and further guided individualized treatment.

## Data Availability

The datasets presented in this study can be found in online repositories. The names of the repository/repositories and accession number(s) can be found in the article/Supplementary Material.

## Abbreviations

CCLE, Cancer Cell Line Encyclopedia; CLs, cancer cell lines; CR, complete response; CSD, stable disease; CTLs, cytotoxic T lymphocytes; DEGs, differentially expressed genes; EMT, epithelial–mesenchymal transition; FPI, ferroptosis index; GC, gastric cancer; GDSC, Genomics of Drug Sensitivity in Cancer; GEO, Gene Expression Omnibus; GO, Gene ontology; GSVA, Gene set variation analysis; ICB, immune checkpoint blockade; KEGG, Kyoto Encyclopedia of Genes and Genomes; PC, principal component; PCA, principal component analysis; PD, progressive disease; ssGSEA, single sample gene set enrichment analysis; PR, partial response; TCGA, the Cancer Genome Atlas; TIDE, Tumor Immune Dysfunction and Exclusion; TME, tumor microenvironment; t-SNE, t-distributed stochastic neighbor embedding.
