# Supplementary figures and images for: Comprehensive Analysis of the Aberrance and Functional Significance of Ferroptosis in Gastric Cancer
Source: Front Pharmacol. 2022 Jul 12;13:919490. doi: 10.3389/fphar.2022.919490 (PMC9315307; doi:10.3389/fphar.2022.919490)

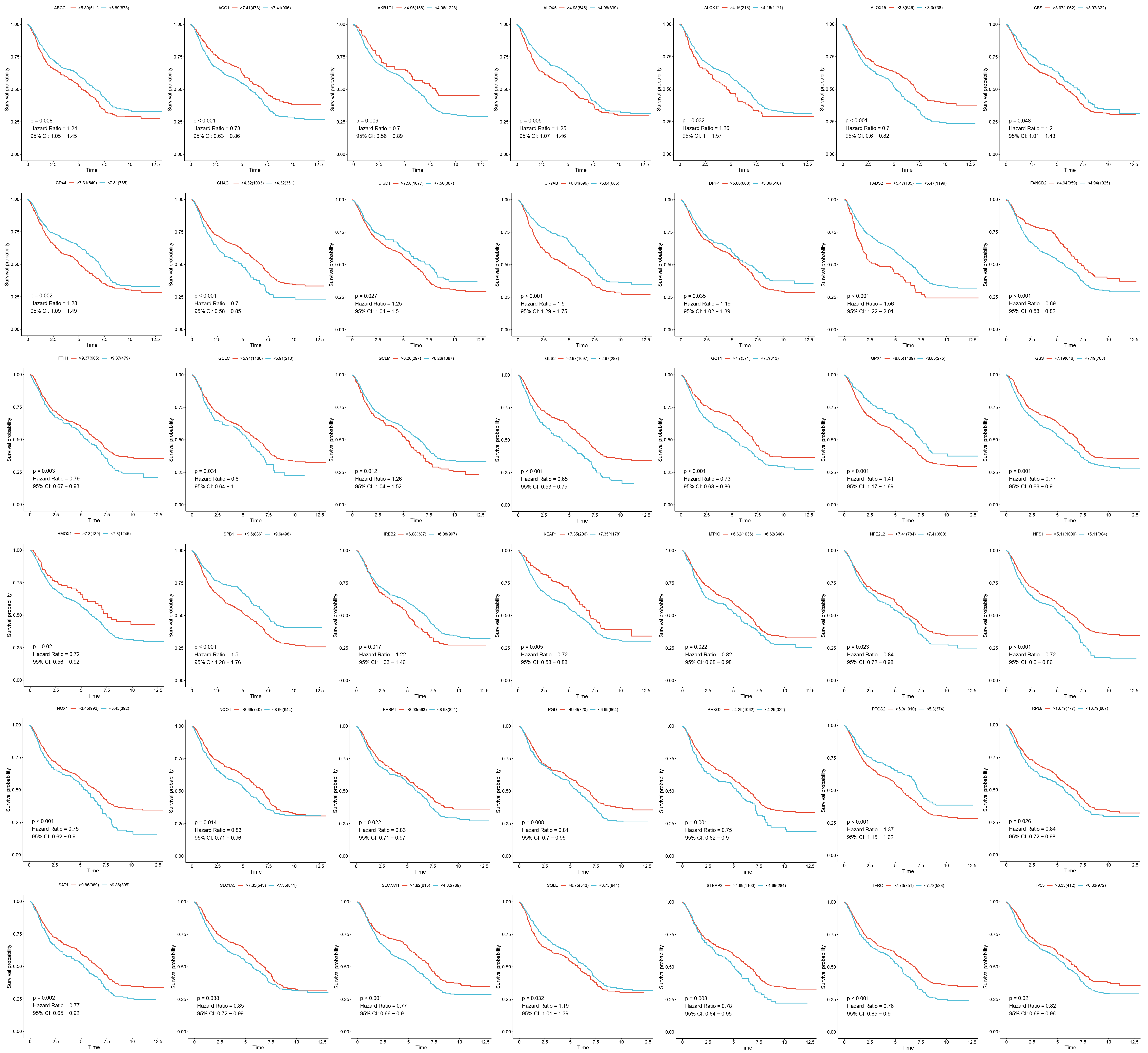

Supplement: Supplementary file 3 [file Image1.TIF]
